# Plasma Phosphorylated-tau181 Is a Predictor of Post-stroke Cognitive Impairment: A Longitudinal Study

**DOI:** 10.3389/fnagi.2022.889101

**Published:** 2022-04-29

**Authors:** Li-Kai Huang, Shu-Ping Chao, Chaur-Jong Hu, Li-Nien Chien, Hung-Yi Chiou, Yu-Chun Lo, Yi-Chen Hsieh

**Affiliations:** ^1^Department of Neurology, Shuang Ho Hospital, Taipei Medical University, New Taipei City, Taiwan; ^2^Dementia Center, Shuang Ho Hospital, Taipei Medical University, New Taipei City, Taiwan; ^3^Graduate Institute of Humanities in Medicine, Taipei Medical University, Taipei, Taiwan; ^4^Taipei Neuroscience Institute, Taipei Medical University, Taipei, Taiwan; ^5^Ph.D. Program for Neural Regenerative Medicine, College of Medical Science and Technology, Taipei Medical University and National Health Research Institutes, Taipei, Taiwan; ^6^Department of Neurology, School of Medicine, College of Medicine, Taipei Medical University, Taipei, Taiwan; ^7^Graduate Institute of Neural Regenerative Medicine, College of Medical Science and Technology, Taipei Medical University, Taipei, Taiwan; ^8^Graduate Institution of Data Science, College of Management, Taipei Medical University, Taipei, Taiwan; ^9^School of Health Care Administration, College of Management, Taipei Medical University, Taipei, Taiwan; ^10^Health Data Analytics and Statistics Center, Office of Data Science, Taipei Medical University, Taipei, Taiwan; ^11^Institute of Population Health Sciences, National Health Research Institutes, Zhunan, Taiwan; ^12^Master Program in Applied Epidemiology, College of Public Health, Taipei Medical University, Taipei, Taiwan; ^13^School of Public Health, College of Public Health, Taipei Medical University, Taipei, Taiwan; ^14^Ph.D. Program in Biotechnology Research and Development, College of Pharmacy, Taipei Medical University, Taipei, Taiwan

**Keywords:** early-onset PSCI, delayed-onset PSCI, p-tau181, ischemic stroke, biomarker

## Abstract

**Introduction:**

Post-stroke cognitive impairment (PSCI) cannot be neglected because it drastically influences the daily life of patients and their families. However, there are no studies exploring the association between preclinical blood biomarkers of neurodegeneration including plasma amyloid-β (Aβ), tau, and brain-derived neurotrophic factor (BDNF) together with the risk of PSCI. This longitudinal study was to investigate whether these blood biomarkers with imaging markers of cerebral small vessel disease can improve the prediction for PSCI. In addition, we also explored the association between blood biomarkers with the trajectories of PSCI.

**Methods:**

Adult patients with first-ever acute ischemic stroke were recruited, and the cognitive and functional abilities of these patients were evaluated. Furthermore, blood biomarkers of neurodegeneration including plasma Aβ-40, Aβ-42, total tau, phosphorylated tau 181 (p-tau181), and BDNF levels and image markers of cerebral small vessel disease were measured. Each patient was followed up at 3 and 12 months at the outpatient department.

**Results:**

Of 136 patients, 40 and 50 patients developed PSCI at 3 and 12 months after stroke, respectively. In functional trajectories, 27 patients did not have PSCI at 3 months but did at 12 months. By contrast, the PSCI status of 17 patients at 3 months was reversed at 12 months. Patients with high-acute plasma p-tau181 had a significantly lower PSCI risk at 3 months (odds ratio [OR] = 0.62, 95% CI = 0.40–0.94, *p* = 0.0243) and 12 months (OR = 0.69, 95% CI = 0.47–0.99, *p* = 0.0443) after adjustment for covariates and image biomarkers. Discrimination and reclassification statistics indicated that the p-tau181 level can improve discrimination ability for PSCI at 3 and 12 months, respectively. In addition, the plasma p-tau181 level was the highest in subjects without PSCI followed by those with delayed-onset PSCI and early-onset PSCI with reversal, whereas the lowest plasma p-tau181 level was found among those with persistent PSCI, showing a significant trend test (*p* = 0.0081).

**Conclusion:**

Plasma p-tau181 is a potential biomarker for predicting early- and delayed-onset PSCI. Future studies should incorporate plasma p-tau181 as an indicator for timely cognitive intervention in the follow-up of patients with stroke.

## Introduction

Cognition decline after stroke is not rare. The clinical course of post-stroke cognitive impairment (PSCI) is not a unitary syndrome but varies from individual to individual (Patel et al., 2002). Cognitive impairment assessment is often performed at 3–6 months after acute stroke to provide sufficient time for delirium resolution and neurological stability. Notably, stroke patients free of early-onset PSCI (3–6 months after stroke) are still at risk of delayed-onset PSCI (>6 months after stroke), which suggests an underlying pathological process beyond 3 months after stroke (Wagle et al., 2011). Many risk factors for PSCI have been proposed based on observational studies, including age, sex, educational attainment, stroke severity, stroke histories, and cardiovascular risk factors—particularly diabetes mellitus and hypertension (Sun et al., 2014). Brain image risk factors include white matter hyperintensities and gray matter and hippocampal volumes loss (Dichgans and Leys, 2017; Mok et al., 2017; Ding et al., 2019). However, there is still a lack of precise blood biomarkers for predicting PSCI.

Accumulation of amyloid-beta (Aβ) peptides and phosphorylated tau (p-tau) in the brain are both key pathological features of the Alzheimer’s disease (Glenner and Wong, 1984; Grundke-Iqbal et al., 1986; Jack et al., 2018; Drummond et al., 2020). Several previous studies implicated plasma Aβ40 and Aβ42 levels as associated with cognitive decline among older adults and stroke patients (Tang et al., 2018; Giudici et al., 2020). Our previous work also showed plasma Aβ42 and tau levels at 3 months were lower in the patients with PSCI at 1 year than in those without PSCI (Chi et al., 2019).

Some cohorts found that plasma tau phosphorylated at threonine 181 (p-tau181) was associated with cognitive decline (Karikari et al., 2020). In addition, brain-derived neurotrophic factor (BDNF) is an important neurotrophin in the adult brain, which can help the brain to repair (Liu et al., 2020). A previous study further found that serum BDNF levels are decreased in the acute phase of stroke, and lower circulating concentrations of BDNF protein are associated with poor long-term functional outcomes (Stanne et al., 2016).

Since these key peptides and proteins play important roles in cognitive performance, and there are few studies to examine the plasma levels of Aβ42, tau, BDNF, and p-tau181 in patients after stroke through longitudinal follow-up, the purpose of this study was to investigate whether these blood with imaging markers can improve the prediction for PSCI. In addition, the association between blood biomarkers with the trajectories of PSCI was also examined.

## Materials and Methods

### Study Participants

Patients aged ≥ 20 years who were admitted to Shuang-Ho Hospital, Taipei Medical University within 7 days of acute ischemic stroke were screened for enrollment eligibility between 2015 and 2018. Patients with known premorbid cognitive impairment, mood disorders, or neurodegenerative diseases that have impaired daily activities were excluded. In order to focus on the cognitive trajectory after stroke, we excluded the major cognitive impairment of stroke *per se*: large infarcts that cause immediate consciousness impairment; strategic infarcts involving the hippocampus or medial frontal cortex; and severe language or physical disabilities that hinder neuropsychological testing. Each patient was evaluated in the hospital within 7 days of the stroke and followed up at the outpatient department at 3 and 12 months post-stroke. The Institutional Review Board of the Taipei Medical University approved the study. Written informed consent was obtained from all patients or their legal guardians.

### Data Collection

Brain magnetic resonance images were obtained once at admission, including T1- and T2-weighted images, T2 fluid-attenuated inversion recovery (T2 FLAIR) images, diffusion-weighted images (DWI), apparent diffusion coefficient (ADC) maps, T2 star-weighted angiography (SWAN), and time-of-flight magnetic resonance angiograms. Acute ischemic brain infarction was confirmed with hyperintensity on DWI with corresponding ADC maps. Visual ratings of white matter hyperintensities (WMHs) were performed by an investigator who was blinded to the clinical details by applying the Fazekas rating scale (Fazekas et al., 1987). Microbleeds were rated as round- or oval-shaped dark blooming signals (2–10 mm in diameter) using the Microbleeds Anatomical Rating Scale (MARS) on the SWAN image and were classified into deep, lobar, and infratentorial categories (Gregoire et al., 2009). Stroke severity was assessed using the National Institute of Health Stroke Scale (NIHSS) at admission. NIHSS is a 15-item impairment scale, each of which scores a specific ability between 0 and 4. Total Score ranges from 0 to 42. The stroke etiological subtype was classified according to The Trial of ORG 10172 in Acute Stroke Treatment (TOAST) classification: (1) large-artery atherosclerosis, (2) cardioembolism, (3) small-vessel occlusion, (4) stroke of other determined etiology, and (5) stroke of undetermined etiology (Adams et al., 1999) by an experienced neurologist who was unaware of the cognition outcomes of patients. A total of two neuropsychologists blinded to patients’ plasma biomarker data conducted cognitive function assessments using the Taiwanese version of the Montreal Cognitive Assessment (MoCA) screening instrument (Tsai et al., 2012) and the Clinical Dementia Rating (CDR) global score, Sum of Boxes (CDR-SB; range: 0–18). The MoCA showed a low ceiling effect with high sensitivity and specificity when used for assessing cognitive impairment after stroke (Pendlebury et al., 2012). The CDR–SB comprises six cognitive and functional domains (memory, orientation, judgment, and problem-solving, community affairs, home and hobbies, and personal care), which yield additional information, particularly regarding mild impairment (Lynch et al., 2006). In this study, we defined PSCI as a CDR-SB > 0 when the patient presented with functional impairment after stroke in either one or more of the six domains. Given the diverse clinical presentation of PSCI, in addition to the memory domain, other cognitive functions must also be assessed (Skrobot et al., 2018). We further classified patients based on the 3 and 12-month CDR-SB assessments to understand the trajectory of PSCI after stroke. Persistent non-PSCI is defined as 3 and 12-month CDR-SB = 0 (*n* = 69), delayed-onset PSCI is CDR-SB = 0 at 3 months while CDR-SB ≥ 0.5 at 12 months (*n* = 27), early PSCI with reversal is 3-month CDR-SB ≥ 0.5 and 12-month CDR-SB = 0 (*n* = 17), and persistent PSCI is CDR-SB ≥ 0.5 at 3 and 12 months (*n* = 23).

### Measurement of Plasma Biomarkers

A total of two 10 ml non-fasting venous blood was collected within 7 days of stroke onset. The blood samples were centrifuged at 1,500 × *g* for 15 min at room temperature and the plasma in the EDTA tube was transferred and aliquoted into 0.5-ml microcentrifuge tubes stored at −80°C until biomarker assays. Plasma Aβ40, Aβ42, total tau, and p-tau181 were analyzed using immunomagnetic reduction (IMR) assays manufactured by MagQu Co. Ltd. (New Protein Analysis Taipei City, Taiwan). Technical details of IMR assays have been described in previous studies (Tang et al., 2018; Yang et al., 2018; Chi et al., 2019). The BDNF was quantified through enzyme-linked immunosorbent assay by using cytokine detection kits (DY248; R&D Systems, Minneapolis, MN, United States) according to the manufacturer’s protocol. Absorbance at 450 nm was measured with a SpectraMax microplate reader (Molecular Devices, San Jose, CA, United States). All the samples were analyzed in duplicate.

### Statistical Analysis

Continuous variables are presented as mean and SD, and data with non-normal distribution are expressed as medians with interquartile ranges. Categorical variables are presented in terms of the frequency with percentage. Univariate logistic regression was used to estimate the odds ratios (ORs) of PSCI at 3 and 12 months based on clinical characteristics and laboratory data. They identified important covariates with borderline significance in the univariate analysis were then verified in the multivariate logistic model by using automatic forward selection methods. Receiver operating characteristic (ROC) analysis was conducted to estimate the performance of plasma biomarkers combined with important covariates for differentiating PSCI risk at 3 and 12 months. In addition, the net reclassification index (NRI) and integrated discrimination improvement (IDI) were computed to evaluate the incremental prognostic value of plasma biomarkers beyond conventional risk factors as well as image biomarkers. All statistical analyses were performed using SAS (version 9.4, Cary, NC, United States). A two-tailed *p* < 0.05 was considered statistically significant.

## Results

**FIGURE 1:**
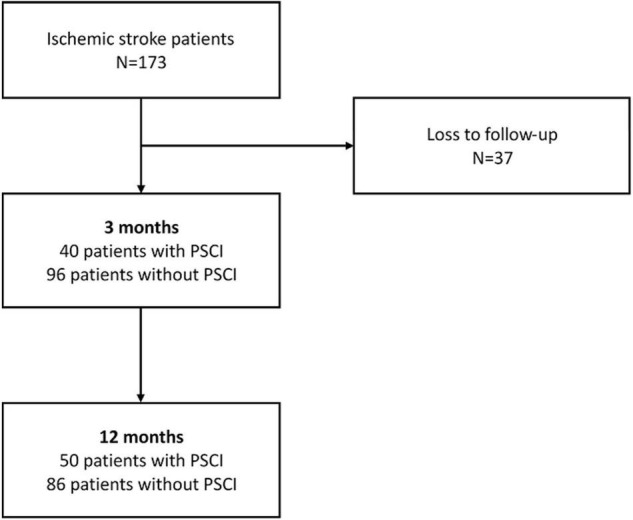
Flowchart of patient enrollment and cognitive function changes during the 12 months’ follow-up.

All 173 participants completed initial clinical and neuropsychological examinations as well as plasma biomarker assays and a brain MRI scan within 7 days after stroke. Each individual was given a standardized treatment scheme during hospitalization. No patient died during the follow-up time of 1 year. Attrition was due to loss to follow-up in 37 patients at 3 months. No significant difference between the baseline profiles of participants involved in this study and those lost in follow-up was found. Data from 136 patients were processed in the final analysis. At 3 months after stroke, 40 patients met the criteria for PSCI according to CDR-SB > 0, whereas at 12 months after stroke, 50 patients had PSCI (Figure 1).

### Characteristics of Study Subjects

Demographic data, brain MRI visual rating scores, and plasma biomarkers are summarized in Table 1. Dividing into two groups according to whether positive of PSCI at 3 months after stroke, the mean ages of patients with and without PSCI were 61.43 ± 12.57 and 57.73 ± 12.66 years, respectively. A total of 40% of patients with PSCI have an education level of >9 years, which is significantly lower than that of patients with non-PSCI. A remarkably high frequency of hypertension was observed among patients with PSCI compared with those without PSCI. Most patients had mild stroke severity, and median NIHSS scores within 7 days for patients with and without PSCI were 4 and 3, respectively. Periventricular white matter Fazeka scale was significantly different between the two groups. MoCA scores at 3 and 12 months were significantly lower in patients with PSCI than in those without PSCI. Furthermore, the plasma p-tau181 level in patients with PSCI (3.19 ± 1.77 pg/ml) was significantly lower than in those without PSCI (4.16 ± 2.18 pg/ml).

**TABLE 1:** Basic characteristics of study subjects with and without PSCI defined at 3 months after stroke.

Variables		With PSCI	Without PSCI	*P*-value
		
Demographics		*N* = 40	*N* = 96	
AGE, y, mean (SD)		61.43 (12.57)	57.73 (12.66)	0.1225
Male, *n* (%)		30 (75.00)	67 (69.79)	0.5406
BMI, kg/m^2^, median (IQR)		25.71 (3.95)	25.68 (4.14)	0.2448
Education > 9 years, *n* (%)		16 (40.00)	58 (60.42)	0.0294
Cigarette smoking, *n* (%)		25 (62.50)	46 (48.42)	0.1347
Alcohol drinking, *n* (%)		4 (10.00)	11 (11.58)	0.7898
**Medical history**				
Hypertension, *n* (%)		36 (90.00)	72 (75.00)	0.0487
Diabetes mellitus, *n* (%)		16 (40.00)	32 (33.33)	0.4585
Dyslipidemia, *n* (%)		31 (77.50)	72 (75.00)	0.7566
**Clinical features**				
Fasting glucose, mg/dL, median (IQR)		111.00 (30.00)	110.00 (29.00)	0.7986
Total cholesterol, mg/dL, median (IQR)		203.00 (66.50)	198.00 (62.00)	0.6652
NIHSS ≤ 7 days, score, median (IQR)		4.00 (3.50)	3.00 (3.00)	0.5064
MoCA at 3 months, score, median (IQR)		22.50 (6.50)	26.00 (5.00)	<0.0001
MoCA at 12 months, score, median (IQR)		23.00 (7.00)	27.00 (5.00)	0.0007
**Plasma biomarkers**				
Aβ 42, pg/mL, median (IQR)		15.54 (2.48)	15.66 (3.18)	0.5763
Aβ 40, pg/mL, median (IQR)		49.45 (7.76)	49.75 (7.22)	0.6824
Aβ 42/40 ratio,%, median (IQR)		32.60 (10.47)	34.56 (11.32)	0.3166
Tau, pg/mL, median (IQR)		18.87 (9.43)	18.64 (13.71)	0.4920
p-tau181, pg/mL, mean (SD)		3.19 (1.77)	4.16 (2.18)	0.0053
BDNF, pg/mL, median (IQR)		732.43 (317.00)	724.97 (387.01)	0.7755
**TOAST**				
Large artery atherosclerosis, *n* (%)		8 (20.00)	17 (17.71)	0.3289
Small vessel occlusion, *n* (%)		24 (60.00)	61 (63.54)	
Cardioembolism, *n* (%)		3 (7.50)	10 (10.42)	
Specific etiology, *n* (%)		3 (7.50)	1 (1.04)	
Undetermined etiology, *n* (%)		2 (5.00)	7 (7.29)	
**Fazekas scale**				
Periventricular white matter	0	4 (10.26)	33 (34.38)	0.0036
	1	12 (30.77)	34 (35.42)	
	2	6 (15.38)	4 (4.17)	
	3	17 (43.59)	25 (26.04)	
Deep white matter lesion	0	5 (12.82)	27 (28.13)	0.1803
	1	17 (43.59)	42 (43.75)	
	2	9 (23.08)	13 (13.54)	
	3	8 (20.51)	14 (14.58)	
**Mirobleed anatomical rating scale**				
Infratentorial score	0	29 (76.32)	81 (84.38)	0.2812
	1	7 (18.42)	8 (8.33)	
	2–4	2 (5.26)	7 (7.29)	
	>4	0 (0)	0 (0)	
Deep score	0	27 (71.05)	76 (79.17)	0.5389
	1	6 (15.79)	10 (10.42)	
	2–4	4 (10.53)	9 (9.38)	
	>4	1 (2.63)	1 (1.04)	
Lobar score	0	25 (65.79)	74 (77.08)	0.0617
	1	3 (7.89)	13 (13.54)	
	2–4	9 (23.68)	7 (7.29)	
	>4	1 (2.63)	2 (2.08)	

*Abbreviations: PSCI, post-stroke cognitive impairment; BMI, body mass index; NIHSS, National Institute of Health Stroke Scale; MoCA, Montreal Cognitive Assessment; Aβ, amyloid-beta; p-tau181, phosphorylated tau 181; BDNF, brain-derived neurotrophic factor; IQR, interquartile range; SD, standard deviation.*

### Univariate and Multivariate Regression Analyses of Post-stroke Cognitive Impairment Risk

Table 2 presents the results of univariate and multivariate logistic regression analyses for patients with and without PSCI at both 3 and 12 months, respectively. For PSCI at 3 months, education level, plasma p-tau181 level, periventricular white matter Fazekas scale, and MARS lobar score were significant factors in the univariate model. These identified important covariates together with hypertension were then verified in the multivariate analysis. After forward selection, education level, hypertension, and plasma p-tau181 level were significantly independent factors of PSCI, showing the patients having an increased p-tau181 level had a significantly lower risk of PSCI at 3 months (OR = 0.62, 95% CI = 0.40–0.94, *p* = 0.0243). Similar findings were found when the patients were followed up for 12 months. Age, periventricular white matter Fazeka scale were significant factors in the univariate model. After forward selection, the plasma p-tau181 level was a significant independent factor of PSCI. For patients with increment of p-tau181 level had a 0.69-fold risk of PSCI at 12 months (95% CI = 0.47–0.99, *p* = 0.0443). However, plasma tau, Aβ42, Aβ40, and BDNF were not predictors of PSCI at 3 or 12 months.

**TABLE 2:** Univariate and multivariate logistic regression analyses for patients with PSCI and without PSCI at 3 and 12 months, respectively.

	3 months				12 months			
Variables	OR (95%CI)	*p*-value	OR*^a^* (95%CI)	*p*-value	OR (95%CI)	*p*-value	OR*^a^* (95%CI)	*p*-value
AGE	1.02 (0.99–1.06)	0.1240			1.03 (1.00–1.06)	0.0261		
Male	1.30 (0.56–3.00)	0.5412			0.90 (0.42–1.94)	0.7947		
Education > 9	0.44 (0.21–0.93)	0.0312	0.27 (0.09–0.81)	0.0191	0.51 (0.25–1.04)	0.0645		
Hypertension	3.00 (0.97–9.30)	0.0571	8.39 (1.44–48.90)	0.0181	0.87 (0.37–2.05)	0.7563		
Diabetes mellitus	1.33 (0.62–2.86)	0.4592			1.58 (0.76–3.26)	0.2135		
Dyslipidemia	1.15 (0.48–2.75)	0.7568			1.02 (0.45–2.31)	0.9563		
Smoking	1.78 (0.83–3.78)	0.1368			1.52 (0.74–3.09)	0.2481		
Drinking	0.85 (0.25–2.84)	0.7900			0.40 (0.11–1.50)	0.1754		
NIHSS ≤ 7 days	1.01 (0.91–1.11)	0.9176			1.01 (0.92–1.10)	0.9206		
**Plasma biomarkers at baseline**								
Tau, pg/mL	0.97 (0.92–1.01)	0.1737			1.00 (0.96–1.04)	0.9822		
Aβ 42, pg/mL	0.98 (0.83–1.16)	0.7846			1.05 (0.90–1.23)	0.5597		
Aβ 40, pg/mL	1.01 (0.93–1.10)	0.7445			1.07 (0.98–1.17)	0.1458		
p-tau181, pg/mL	0.63 (0.43–0.91)	0.0151	0.62 (0.40–0.94)	0.0243	0.72 (0.51–1.02)	0.0640	0.69 (0.47–0.99)	0.0443
BDNF, pg/mL	1.00 (1.00–1.00)	0.6768			1.00 (1.00–1.00)	0.1198		
**Fazekas scale**								
Periventricular white matter	1.66 (1.20–2.31)	0.0024			1.45 (1.07–1.97)	0.0154		
Deep white matter lesion	1.44 (0.99–2.10)	0.0547			1.09 (0.77–1.55)	0.6323		
**Mirobleed anatomical rating scale**								
Intratentorial score	1.43 (0.95–2.15)	0.0832			1.49 (0.94–2.36)	0.0916		
Deep score	1.42 (0.99–2.03)	0.0549			1.31 (0.94–1.82)	0.1109		
Lobar score	1.60 (1.08–2.37)	0.0190			1.34 (0.97–1.86)	0.0791		

*^a^Multiple logistic regression model using the automatic forward selection. Abbreviations: PSCI, post-stroke cognitive impairment; NIHSS, National Institute of Health Stroke Scale; Aβ, amyloid-beta; p-tau181, phosphorylated tau 181; BDNF, brain-derived neurotrophic factor.*

### Predictive Accuracy of p-tau181 for Assessing Post-stroke Cognitive Impairment

**FIGURE 2:**
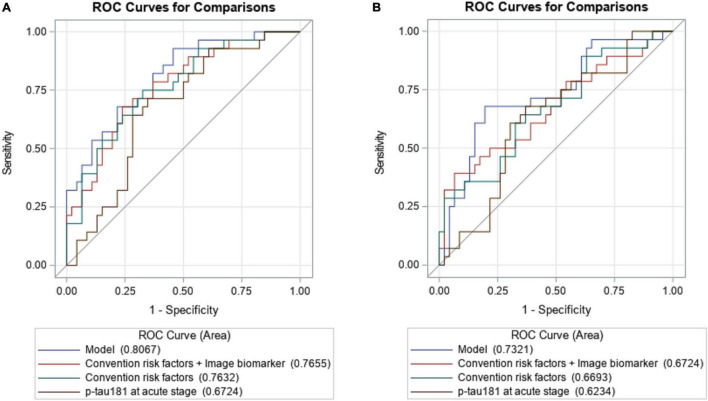
The area under the receiver operating characteristic of the p-tau181 predicting PSCI at 3 **(A)** and 12 months **(B)**, respectively, when compared to conventional risk factors with image markers.

To examine the effect of p-tau181 on PSCI at 3 and 12 months, respectively, in addition to conventional risk factors, discrimination and reclassification statistics were calculated. Figure 2A illustrates the area under the curve (AUC) of the model at 3 months based only on conventional risk factors was 0.7632 (95% CI = 0.6520–0.8744), but it slightly increased to 0.7655 (95%CI = 0.6549–0.8761) when image biomarkers were added. After integrating plasma p-tau181 level, the performance has been greatly improved, which achieved good discrimination ability (AUC = 0.8067; 95% CI = 0.7066–0.9067). Similar findings were also found when analyzing the predictive effects at 12 months (Figure 2B). According to the NRI and IDI indexes, adding the p-tau181 level to the model containing conventional risk factors and image biomarkers significantly improves the measure of reclassification and discrimination for PSCI at 3 and 12 months (Table 3).

**TABLE 3:** Discrimination and reclassification statistics of p-tau181 level for patients with PSCI at 3 and 12 months, respectively.

Clinical outcomes	Model	NRI index			IDI index		
		Estimate	95%CI	*p*-value	Estimate	95%CI	*p*-value
PSCI at 3 month	Conventional model						
	Conventional model + image biomarker	0.369	(0.002–0.735)	0.0522	0.035	(0.004–0.067)	0.0289
	Conventional model + image biomarker + p-tau181 level	0.786	(0.417–1.155)	0.0010	0.144	(0.074–0.214)	<0.0001
PSCI at 12 months	Conventional model						
	Conventional model + image biomarker	0.235	(−0.111–0.582)	0.1884	0.016	(−0.002–0.034)	0.0890
	Conventional model + image biomarker + p-tau181 level	0.730	(0.326–1.134)	0.0023	0.086	(0.028–0.145)	0.0040

Conventional risk factors included age, gender, education, hypertension, diabetes mellitus, and NIHSS ≤ 7 days. Abbreviations: PSCI, post-stroke cognitive impairment; NRI, net reclassification index; IDI, integrated discrimination improvement; p-tau181, phosphorylated tau 181.

### Association Between Plasma Biomarkers and Different Persistent Cognitive Impairment Status

Plasma biomarkers were analyzed according to the trajectory of cognitive impairment status defined by the CDR-SB at 3 and 12 months. Table 4 presents the levels of plasma tau, Aβ42, Aβ40, Aβ42/40 ratio, p-tau181, and BDNF among the four subgroups. The results indicated that only plasma p-tau181 levels differed among the four groups, with the highest level in the persistent non-PSCI group (4.40 ± 1.77 pg/ml) followed by the delayed-onset PSCI (3.65 ± 1.41 pg/ml), early PSCI with reversal (3.36 ± 1.38 pg/ml) groups, and the lowest level was in the persistent PSCI group (3.12 ± 0.78 pg/ml), showing a significant trend test (*p* = 0.0081).

**TABLE 4:** Association between plasma biomarkers and different persistent cognitive impairment statuses according to CDR-SB at 3 and 12 months.

Plasma biomarkers	Persistent non-PSCI (*n* = 69)	Delayed-onset PSCI (*n* = 27)	Early PSCI with reversal (*n* = 17)	Persistent PSCI (*n* = 23)	*p*-value
Aβ 42, pg/mL, median (IQR)	15.65 (3.26)	16.04 (3.86)	15.84 (2.86)	15.40 (2.29)	0.7145
Aβ 40, pg/mL, median (IQR)	49.58 (7.03)	49.99 (8.68)	48.22 (5.01)	51.49 (8.75)	0.1796
Aβ 42/40 ratio,%, median (IQR)	0.35 (0.10)	0.33 (0.11)	0.33 (0.17)	0.32 (0.12)	0.2213
Tau, pg/mL, median (IQR)	18.43 (11.62)	19.69 (16.03)	19.63 (9.33)	18.54 (7.38)	0.6076
p-tau181, pg/mL, mean (SD)	4.40 (1.77)	3.65 (1.41)	3.36 (1.38)	3.12 (0.78)	0.0081
BDNF, pg/mL, median (IQR)	776.52 (359.35)	639.77 (378.88)	750.62 (261.49)	729.74 (402.71)	0.9267

*Abbreviations: PSCI, post-stroke cognitive impairment; CDR-SB, Clinical Dementia Rating global score, Sum of Boxes; Aβ, amyloid-beta; p-tau181, phosphorylated tau 181; BDNF, Brain-derived neurotrophic factor; IQR, interquartile range; SD, standard deviation.*

## Discussion

This study investigated 136 patients with acute ischemic stroke and followed them up for consequential cognitive function trajectory for 1 year. The results demonstrated that a high plasma p-tau181 level in the acute post-stroke stage is related to a low PSCI risk at 3 (OR = 0.62, 95% CI = 0.40–0.94, *p* = 0.0243) and 12 (OR = 0.69, 95% CI = 0.47–0.99, *p* = 0.0443) months. Integrating p-tau181 to the model containing conventional risk factors (age, education, hypertension, diabetes mellitus, and NIHSS score at ≤7 days) and image biomarker significantly improves prediction.

Cognitive improvement after stroke often occurs within 6 months, and early recognition of PSCI is necessary for assessing the need for rehabilitation (Turunen et al., 2018). In the case of cerebral ischemia, hyperphosphorylated tau protein may play a protective role by promoting β-catenin and other proteins to inhibit cell apoptosis, suggesting that neurons survive apoptotic attacks and achieve self-repair (Wang et al., 2010). Phosphorylation of tau results in reduced binding to microtubules and potentially enhances plasticity, as observed during brain development, which is also important in neural repair after stroke (Alonso et al., 2001, 2006; Wang et al., 2007; Morris et al., 2011). In Alzheimer’s disease (AD), plasma p-tau181 is a prognostic and confirmatory biomarker (Bateman et al., 2020). Hyperphosphorylated tau protein forms paired helical filaments and progressively aggregates to form the main component of neurofibrillary tangles in AD pathology (Pevalova et al., 2006). However, unlike in AD pathology, neurofibrillary tangles are rarely seen in the brain after minor stroke. Exceptional case series of NFTs in the ipsilateral basal nucleus of Meynert (BNM) associated with a massive cerebral infarct in the MCA territory or a putaminal hemorrhage, speculated to form within the 5–10 years after stroke onset (Hatsuta et al., 2019). Studies of cerebrospinal fluid (CSF) samples from patients with acute stroke showed that CSF p-tau did not increase, whereas CSF total tau increased and returned to normal levels at 3–5 months after stroke, suggesting different pathogenic processes from AD (Hesse et al., 2001; Hjalmarsson et al., 2014; Hagberg et al., 2020). Therefore, the diagnostic criteria for vascular cognitive disorders from the International Society of Vascular Behavioral and Cognitive Disorders statement suggest excluding CSF p-tau when diagnosing vascular cognitive impairment in research (Sachdev et al., 2014), whereas a persistent increase in CSF p-tau is considered a defining biomarker of AD in the National Institute on Aging—Alzheimer’s Association Research Framework (Albert et al., 2011). In addition, plasma p-tau181 predicted PSCI in a dose-dependent manner in our longitudinal cohort study, and the p-tau181 level was the highest in the persistent non-PSCI group and the lowest in the persistent PSCI group. A protective role for tau phosphorylation at threonine 181 could facilitate its binding to exosomes and the release of excess tau (Avila et al., 2012). Further studies are warranted to understand the mechanism underlying this finding.

Previous studies have revealed that plasma Aβ42/40 ratios are surrogate biomarkers of cortical Aβ deposition (Fandos et al., 2017). High-plasma concentrations of Aβ40, especially when combined with low concentrations of Aβ42, indicate an increased dementia risk (van Oijen et al., 2006). A previous publication by our group using the same cohort but few participants showed Aβ42 and tau levels at 3 months were lower in the patients with PSCI at 1 year than in those without PSCI, which may reveal AD pathology one mechanism of PSCI development after 3 months of stroke and decreased levels of plasma tau could be explained by its association with the decreased plasma Aβ42 levels (Chi et al., 2019). In this study, Aβ42, Aβ40, and Aβ42/40 ratios were not different between patients with and without PSCI, which suggests the involvement of additional processes other than amyloid pathology. In addition, BDNF was found to be an indicator of long-term functional outcomes after ischemic stroke, although the additional predictive value of BNDF was modest according to clinical data (Stanne et al., 2016). Our data showed that there was no significant difference in circulating BDNF levels regardless of whether the patient had PSCI or not, which may be due to its limited impact on cognitive outcomes. In this study, a low-education level, hypertension, and pre-existing periventricular white matter disease were also the major risk factors for PSCI at 3 months. This finding is consistent with those of previous studies (Zhou et al., 2005; Sun et al., 2014; Ding et al., 2019).

Clinical Dementia Rating global score, Sum of Boxes was adopted to define PSCI in our study. Given the diverse clinical presentation of PSCI, not only memory but also other cognitive domains should be evaluated (Skrobot et al., 2018). The global CDR is weighted more on memory dysfunction, whereas CDR-SB is weighted equally for all domains (Wyman-Chick and Scott, 2015). In addition, CDR-SB has been considered an effective and reliable assessment method, which combines two sets of questions, one set is for the insider and the other is for the subject. This implies the use of CDR-SB to define the PSCI has a clinically significant impact on the follow-up after stroke. In our study, none of the patients had dementia before the stroke. However, the prevalence of PSCI at 3 and 12 months after stroke was 29.4 and 36.8%, respectively, during the longitudinal follow-up. The reported prevalence varied among previous studies depending on divergent estimates of PSCI according to the population under study and the methods of defining PSCI, suggesting a need for diagnosis criteria consensus (del Ser et al., 2005; Abzhandadze et al., 2019).

Notably, in our cohort, 27 patients without PSCI at 3 months developed PSCI at 12 months after stroke (late PSCI: 19.9%), and 17 patients reversed from PSCI at 3 months to non-PSCI at 12 months (reversal: 42.5%). Our findings further revealed that a dose-dependent trend of plasma p-tau181 level existed among patients with various persistent cognitive impairment statuses, with the highest level in the non-PSCI group followed by the early PSCI with a reversal and delayed-onset PSCI groups; the lowest level was in the persistent PSCI group, which implicated the protective effect on longitudinal cognitive function after a minor ischemic stroke.

This study has several limitations. First, there was no formal test conducted for measuring baseline cognitive function before stroke in our participants. However, patients with known cognitive impairment or neurodegeneration that impaired daily activities before stroke were excluded based on their medical histories at the screening phase. In addition, this study adopted the CDR-SB to define PSCI in order to distinguish functional changes after stroke since we relied on informant-based evidence rather than performance-based tests. Second, the severity of stroke in the patients in this study is relatively small and mainly involves small vessel diseases which might influence the generalizability of the findings to large brain infarction or intracranial hemorrhage. Future studies will be needed to elucidate the natural course of other types of brain insults. Third, in our study, no further AD diagnosis was performed; however, previous findings indicated that after ischemia with reperfusion in the brain, secondary neurodegeneration of AD type may occur (Pluta, 2000). Further longitudinal studies regarding biomarkers are needed to distinguish between PSCI and AD processes.

In conclusion, PSCI is not uncommon in the population with minor stroke. Using plasma p-tau181 as a surrogate biomarker for predicting early- and delayed-onset PSCI is helpful in interventional studies and clinical follow-up.

## Data Availability Statement

The original contributions presented in the study are included in the article/supplementary material, further inquiries can be directed to the corresponding authors.

## Ethics Statement

The studies involving human participants were reviewed and approved by the Institutional Review Board of Taipei Medical University. The patients/participants provided their written informed consent to participate in this study.

## Author Contributions

L-KH wrote the manuscript with support from Y-CH. Y-CH, S-PC, and L-NC verified the analytical methods. C-JH and H-YC conceived the study and were in charge of overall direction and planning. Y-CL, S-PC, and L-NC aided in interpreting the results and worked on the manuscript. All authors discussed the results and contributed to the final manuscript.

## Conflict of Interest

The authors declare that the research was conducted in the absence of any commercial or financial relationships that could be construed as a potential conflict of interest.

## Publisher’s Note

All claims expressed in this article are solely those of the authors and do not necessarily represent those of their affiliated organizations, or those of the publisher, the editors and the reviewers. Any product that may be evaluated in this article, or claim that may be made by its manufacturer, is not guaranteed or endorsed by the publisher.
